# Arterial Injury to the Profunda Femoris Artery following Internal Fixation of a Neck of Femur Fracture with a Compression Hip Screw

**DOI:** 10.1155/2013/181293

**Published:** 2013-12-17

**Authors:** Simon Craxford, Michael Gale, Kimberly Lammin

**Affiliations:** Department of Orthopaedics, Royal Derby Hospital, Uttoxeter Road, Derby DE22 3NE, UK

## Abstract

We report the case of an 82-year-old woman who developed extensive proximal thigh swelling and persistent anaemia following internal fixation of an extracapsular neck of femur fracture with a dynamic hip screw (DHS). This was revealed to be a pseudoaneurysm of a branch of profunda femoris artery on angiography. Her case was further complicated by a concurrent pulmonary embolism (PE). She underwent endovascular coil embolisation of the pseudoaneurysm. An IVC filter was inserted and the patient was fully anticoagulated once it had been ensured that there was no active bleeding. In this case, we review the potential for anatomical variations in the blood supply to this region and discuss treatment options for a complicated patient. We recommend that a pseudoaneurysm should be part of a differential diagnosis for postoperative patients with anaemia refractory to blood transfusion so as not to miss this rare but potentially serious complication.

## 1. Introduction

Pseudoaneurysm of the profunda femoris artery is a rare complication following fracture of the femoral neck [1–3]. The pseudoaneurysm may form either as a result of arterial injury sustained at the time of fracture or iatrogenically as a consequence of internal fixation [1]. Cases of pseudoaneurysm following arthroscopy [4, 5], application of an external fixator [2, 6], fracture [7], arthrodesis [8], and internal fixation [1–3] have all been previously reported. We present a case of pseudoaneurysm of a branch of the profunda femoris artery following internal fixation of a neck of femur fracture with a compression hip screw (CHS). The management of the patient in this case was complicated by a concurrent pulmonary embolism (PE). This case reminds us of the possibility of pseudoaneurysm as a diagnosis in the patient with postoperative anaemia and also demonstrates a successful method of treatment in the face of a coexisting PE.

## 2. Case Presentation

**Figure 1:**
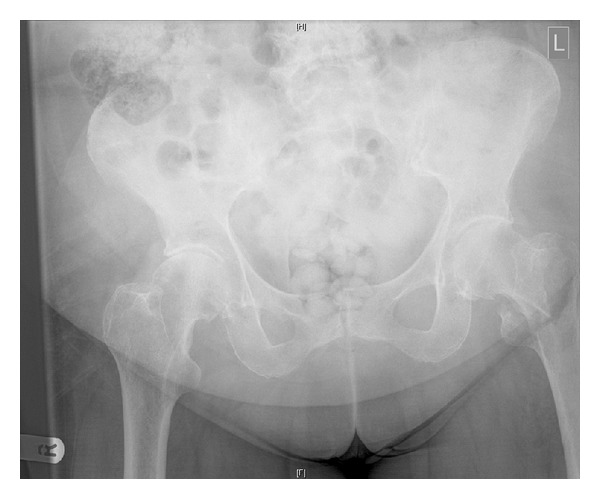
Preoperative AP pelvis demonstrating a left sided intertrochanteric neck of femur fracture.

An 82-year-old woman presented to the emergency department with hip pain and an inability to weight bear following a simple mechanical fall at home. She had a past medical history of chronic kidney disease following curative right total nephrectomy for renal cell carcinoma several years before but was otherwise in good health. On examination her leg was shortened and externally rotated. AP and lateral radiographs of her hip confirmed an extracapsular neck of femur fracture, as shown in Figure 1. After discussion with the patient and her family, consent was taken for internal fixation of the fracture using a dynamic hip screw. Preoperative blood workup revealed a mild anaemia (Hb of 110 g/L) and chronic kidney disease stage 3.

**Figure 2:**
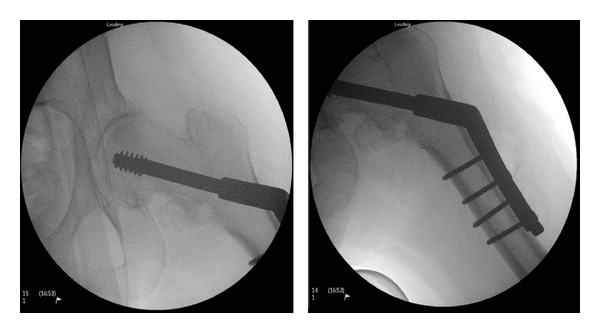
Intraoperative radiographs taken using image intensifier showing a satisfactory position of a compression hip screw.

Surgery was performed on the following day's trauma list by a registrar under image intensifier guidance, as shown in Figure 2. The procedure was supervised by a consultant surgeon. The procedure appeared to be routine with no intraoperative complications noted. The following day the patient had a persistent tachycardia with low oxygen saturations on arterial blood gas sampling. A NM V/Q SPECT (ventilation/perfusion) scan was arranged which revealed a mismatch in the midzone of the left lung, consistent with a pulmonary embolism. After review by the medical team she was started on treatment dose enoxaparin.

**Figure 3:**
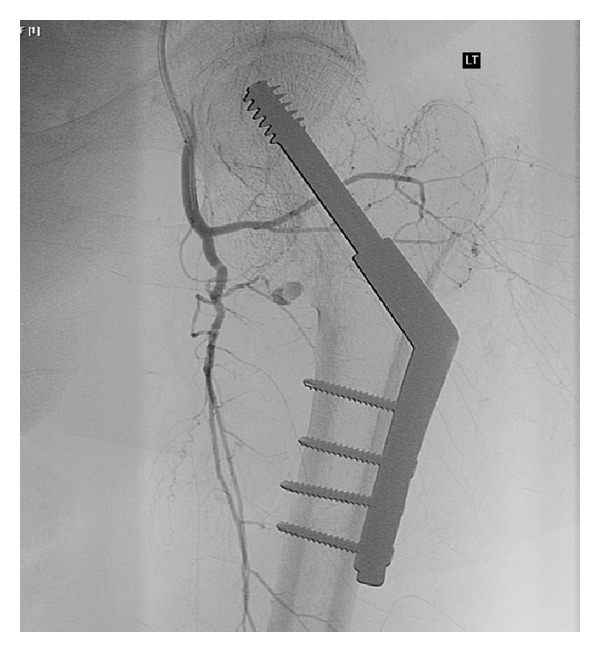
Angiogram demonstrating a pseudoaneurysm of a branch of the profunda femoris artery.

**Figure 4:**
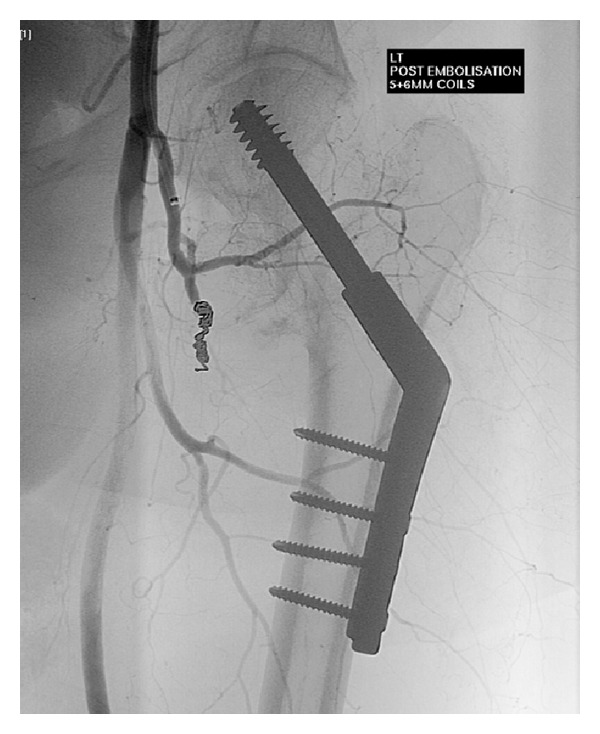
Following endovascular coil embolisation. The angiogram shows no filling of the pseudoaneurysm.

Over the next week she had episodes of intermittent tachycardia and low blood pressure. Her haemoglobin fell from 110 g/L to 80 g/L despite a 3 unit red blood cell transfusion. There were no signs of external bleeding and the surgical wound was clean and dry. Several days after surgery she developed extensive proximal thigh bruising over a 24-hour period, raising the possibility of bleeding from around the fracture site. CT angiography demonstrated a large left thigh haematoma extending along the femoral sheath and a small area of active extravasation of contrast relating to a branch of the profunda femoris artery. Angiography was performed demonstrating a pseudoaneurysm arising from a branch of the proximal profunda corresponding to the abnormality seen on the preceding CT angiogram, as shown in Figure 3. The small supplying vessel was catheterised with difficulty using a microcatheter but a stable position could not be achieved here for embolisation. Therefore the main vessel was embolised at this point using 5 mm and 6 mm 0.035 coils, with preservation of large profunda branches above and below. Follow-up angiography revealed no further active haemorrhage of filling of the pseudoaneurysm, as shown in Figure 4. A Cook Celect retrievable IVC filter was deployed within the infrarenal vena cava. After several days of no further bleeding, warfarin was commenced and the patient was discharged to a community rehabilitation facility. While the pseudoaneurysm did delay her discharge and rehabilitation, she has progressed to make a full recovery.

## 3. Discussion

Pseudoaneurysm following hip surgery is a rare complication. While a superficial pseudoaneurysm may present as a palpable pulsatile mass, a deep pseudoaneurysm may only be detected on further imaging [1–3]. Previous cases of pseudoaneurysm have been reported following both fracture of the neck of the femur [1] and subsequent internal fixation, for example, by CHS fixation [9]. It may be more common if the lesser trochanter is displaced [10]. In our case, it is not clear as to when the pseudoaneurysm initially occurred. Previous papers have identified some technical risk factors specific to DHS fixation. It has been suggested that proper placement of the retractors, using a shorter drill or drill guard, accurate screw length, and a shorter side-plate DHS should be used to reduce the risk of iatrogenic pseudoaneurysm formation [9].

Several management strategies for treating a pseudoaneurysm have been documented in detail in the literature. While some may spontaneously thrombose [11] the usual treatment consists of either surgical or radiological intervention, especially if the patient is unstable or shows signs of active bleeding [12]. Endovascular therapies include endovascular stent insertion [13], thrombin injection [14], or, as in our case, endovascular coil embolisation [15]. Without timely management, a pseudoaneurysm may continue to enlarge or eventually rupture, potentially exposing the patient to severe haemorrhage [16]. This was especially relevant in our patient, who likely suffered greater blood loss from her pseudoaneurysm due to anticoagulation for PE. In this case, her anticoagulation was temporally reversed to allow endovascular embolisation. In patients with a high risk of recurrent PEs but in whom anticoagulation is contraindicated, an IVC filter should be considered [17].

It is important to be aware of both the normal arterial anatomy and the potential for variants. The profunda femoris usually branches from the femoral artery soon after its origin. It then travels between pectineus and adductor longus, running on the posterior aspect of adductor longus. Important branches include the medial and lateral femoral circumflex arteries, which supply the head of the femur and several perforating branches. Several cadaveric and radiological studies have reported variations in the origin and course of profunda femoris and its branches. [18–20]. In a cadaveric study, Prakash et al. reported a wide variance in origin of the medial and lateral femoral circumflex arteries. In the study, 32% of cadaveric medial femoral circumflex arteries originated from the femoral artery and not the profunda femoris. In 18% of cadavers, the lateral femoral circumflex artery also originated directly from the femoral artery. In a cadaveric study by Perera, it was reported that there is a distal migration of the level of origin of the deep femoral artery when either one or both circumflex femoral arteries arise from the femoral artery, rather than from profunda femoris. Without an appreciation for this highly variable arterial course, a surgeon may be falsely reassured that they are far from important arterial structures.

In conclusion, a pseudoaneurysm is a rare complication of surgery to the hip. It may present early or late in the postoperative period and needs to be part of a differential diagnosis for a persistently anaemic postoperative patient. There is a wide variance in both the origin of the profunda femoris and its major branches. Due to the rarity of this complication and the fact that visible bruising may take several days to develop, it may be easily missed or diagnosed late, as in our case. The management of such cases may be challenging if the patient concurrently experiences postoperative complications, such as DVT or PE. The treatment of these complications, anticoagulation, will exacerbate bleeding from a pseudoaneurysm. In these cases a multidisciplinary approach involving surgical, medical, and interventional radiology teams is needed if the correct diagnosis and treatment are to be achieved.
